# Regulation of TGF-β signalling by *Fbxo11*, the gene mutated in the *Jeff *otitis media mouse mutant

**DOI:** 10.1186/1755-8417-2-5

**Published:** 2009-07-06

**Authors:** Hilda Tateossian, Rachel E Hardisty-Hughes, Susan Morse, Maria R Romero, Helen Hilton, Charlotte Dean, Steve DM Brown

**Affiliations:** 1 MRC Mammalian Genetics Unit, Harwell, OX11 0RD, UK

## Abstract

**Background:**

*Jeff *is a dominant mouse mutant displaying chronic otitis media. The gene underlying *Jeff *is *Fbxo11*, a member of the large F-box family, which are specificity factors for the SCF E3 ubiquitin ligase complex. *Jeff *homozygotes die shortly after birth displaying a number of developmental abnormalities including cleft palate and eyes open at birth. TGF-β signalling is involved in a number of epithelial developmental processes and we have investigated the impact of the *Jeff *mutation on the expression of this pathway.

**Results:**

Phospho-Smad2 (pSmad2) is significantly upregulated in epithelia of *Jeff *homozygotes. Moreover, there was a significant increase in nuclear localization of pSmad2 in contrast to wild type. Mice heterozygous for both *Jeff *and *Smad2 *mutations recapitulate many of the features of the *Jeff *homozygous phenotype. However, tissue immunoprecipitations failed to detect any interaction between *Fbxo11 *and *Smad2*. Fbxo11 is known to neddylate p53, a co-factor of pSmad2, but we did not find any evidence of genetic interactions between *Jeff *and *p53 *mutants. Nevertheless, p53 levels are substantially reduced in *Jeff *mice suggesting that Fbxo11 plays a role in stabilizing p53.

**Conclusion:**

Overall, our findings support a model whereby *Fbxo11*, possibly via stabilization of p53, is required to limit the accumulation of pSmad2 in the nucleus of epithelial cells of palatal shelves, eyelids and airways of the lungs. The finding that *Fbxo11 *impacts upon TGF-β signalling has important implications for our understanding of the underlying disease mechanisms of middle ear inflammatory disease.

## Background

Otitis media (OM), inflammation of the middle ear, is the most common cause of hearing impairment in children, potentially causing language delays and learning and behavioural disruption [1,2]. A significant number of children with acute OM will go on to develop OM with effusion or chronic OM. The high prevalence of the disease, coupled with its recurrent and chronic nature, accounts for the large number of tympanostomies, the insertion of ventilation tubes or 'grommets' in the tympanic membrane, undertaken in affected children. OM is still the most common cause of surgery in children in the developed world. However, this and other treatments are largely ineffective.

There is evidence from studies of the human population that there is a significant genetic component predisposing to recurrent or chronic OM [3-5], yet little is known about the underlying genetic pathways involved. From a deafness screen as part of the MRC Harwell mouse mutagenesis programme [6] we have identified two novel dominant mutants, *Jeff *and *Junbo*, which develop a conductive deafness due to a chronic suppurative OM [7-9]. Both these mutants represent the first models for chronic forms of middle ear inflammatory disease in humans, and both of these mutants have now been cloned [8,9].

The gene underlying the *Jeff *mutant was identified as *Fbxo11*, a member of the F-box family [8]. The *Jeff *mutant carries a non-conservative glutamine to leucine change at amino acid 491. F-box proteins function as part of an SCF (SKP1-cullin-F-box) E3 protein ligase complex, recognizing and binding phosphorylated proteins and promoting their ubiquitination and degradation [10,11]. However, the substrate of Fbxo11 is unknown. It has been demonstrated that *Caenorhabditis elegans *DRE-1, an orthologue of human FBXO11, and the SKP1-like homologue SKR-1 function as part of an E3 ligase complex, as does its human counterpart [12]. There is also evidence that FBXO11 has arginine methyltransferase activity, catalyzing arginine methylation, but with a structure different from all other known protein arginine methyltransferases (PRMTs) [13]. PRMT activity was not however detected for DRE-1 [12]. Recently it has been demonstrated that FBXO11 can function as a Nedd8-ligase for the tumour suppressor protein p53, promoting the neddylation of p53 and inhibiting its transcriptional activity [14]. p53 is a partner of Smad2 in the activation of multiple transforming growth factor β (TGF-β) target genes [15].

We previously reported that mice homozygous for the *Jeff *mutation die within a few hours of birth [8]. Newborn *Jeff *homozygotes have cleft palate, facial clefting, impairment of respiratory function and an eyes-open at birth (EOB) phenotype [8]. TGF-β signalling has been shown to be involved in all of these processes [16-18]. For these reasons it will be important to understand the role of *Fbxo11 *in mouse developmental processes and in particular the impact of mutations on the TGF-β signalling pathway.

The TGF-β superfamily is composed of a large number of cytokines involved in a variety of cellular processes such as proliferation, differentiation, epithelial mesenchymal transformation and apoptosis [19,20]. They mediate their effects from membrane to nucleus through combinations of type I and type II serine/threonine kinase receptors (TGFβR-I and TGFβR-II) and their downstream effectors, Smad proteins. Certain receptor-regulated Smads (R-Smads) become phosphorylated by activated type I receptors and form a heteromeric complex with a common-partner Smad4. Once formed, this R-Smad/Smad4 complex translocates to the nucleus and, in conjunction with other nuclear cofactors, regulates the transcription of target genes [19]. Two different Smad signalling branches have been described. The TGF-β sub-family ligands TGF-β, Activins and Nodals, are transduced by Smad2 and Smad3. In contrast, BMP sub-family ligands are transduced by Smad1, Smad5 and Smad8 [19].

TGF-β signalling is controlled by many mechanisms, including ubiquitin-mediated proteosomal degradation [19,21,22]. A key component of ubiquitination, the ubiquitin ligase (E3), controls the specificity and timing of Smad ubiquitination. The E3 ubiquitin ligases, Smad ubiquitination-related factor 1 and 2 (Smurf1 and 2), have been identified as regulators of TGF-β signalling targeting Smad1 for ubiquitination and degradation [23,24]. At higher expression levels, Smurf2 also lowers protein levels of Smad2, but not Smad3 [24]. In addition to regulation of steady-state levels of R-Smads, the ubiquitin-proteosome pathway is also involved in the degradation of activated R-Smads [21,22]. Smurf2 exhibits higher binding affinity to activated Smad2 upon TGF-β stimulation, and Smurf2 is a candidate E3 ligase for activated Smad2 degradation [25]. Roc1, a component of an SCF complex, interacts and promotes degradation of activated Smad3 [26]. MFB-1, a novel F-box-type ubiquitin ligase, negatively regulates Dauer formation in *C. elegans *by modulating DAF-7/TGF-β-like signalling pathway [27]. More recently, Arkadia, a RING-domain E3 ubiquitin ligase, has been shown to interact with and ubiquitinate phospho-Smad2/3 [28]. At the same time, Arkadia enhances transcription, thus coupling turnover of phospho-Smad2/3 to activity.

We have set out to explore the role of *Fbxo11 *in development and to relate the *Jeff *homozygous mutant phenotype to the underlying mechanisms of *Fbxo11 *function. Given the findings of a palatal and EOB phenotype in *Jeff *mice and the role of TGF-β signalling in these processes, we have focused our studies on salient members of the TGF-β family signalling pathway and demonstrated that pSmad2 is upregulated in the epithelia of *Jeff *homozygotes. Moreover, we have utilized compound mutants to assess genetic interactions and throw light on the genetic pathways affected. Mice heterozygous for both *Jeff *and *Smad2 *mutations recapitulate the *Jeff *homozygote phenotype in the palate and lungs. However, we failed to detect any interaction between *Fbxo11 *and *Smad2*, suggesting they are indirect partners in the development of these tissues. Our observations support a model whereby in palate, eyelid and lung *Fbxo11*-dependent modification is required to limit the accumulation of pSmad2 in the nucleus of epithelial cells of palatal shelves, eyelids and airways of the lungs. Overall, we conclude that *Fbxo11 *is involved with the regulation of TGF-β signalling, a finding that has implications for the chronic middle ear inflammatory phenotype that is observed in *Jeff *heterozygotes.

## Results

*Jeff *homozygote mutant mice develop a variety of epithelial developmental abnormalities, including palatal and EOB phenotypes. We used immunohistochemistry (IHC) to study protein expression and localization of members of the TGF-β signalling pathway (TGFβ-3, TGFβR-I, Smad2, Smad3 and Smad4) in *Jeff *mutants in both palatal and eye tissues at relevant developmental stages. Given the apparent involvement of Fbxo11 in epithelial development, we also investigated the expression of these TGF-β signalling pathway members in lung development.

### Palatal expression in Jeff mutants

**Figure 1:**
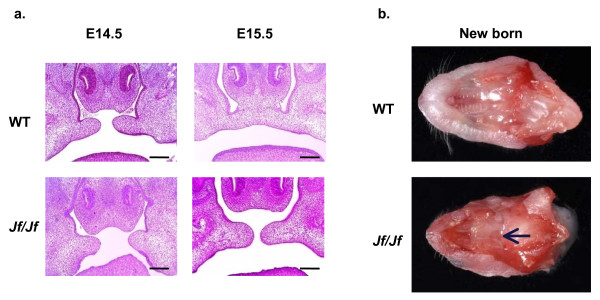
**Cleft palate phenotype**. **a**. Coronal sections through the palate of E14.5 (before the fusion) and E15.5 (after the fusion) wild-type (WT) and homozygote (*Jf/Jf*) embryos, haematoxylin-eosin stained. Scale bars 200 μm. **b**. Cross-sections of heads showing secondary palate of a wild-type (WT) newborn mouse with fused palate and a homozygote (*Jf/Jf*) newborn mouse with a cleft (arrow).

**Figure 2:**
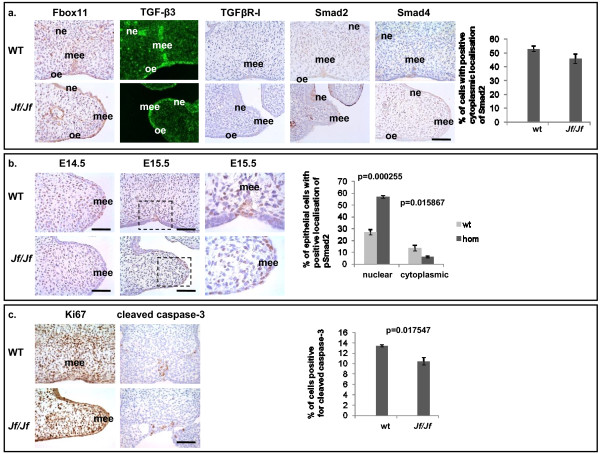
**Immunolocalization in palate tissue**. **a**. Coronal sections through the palate of E15.5 wild-type (WT) and homozygote (*Jf/Jf*) embryos, immunohistochemically stained with antibodies against Fbox11, TGF-β3, TGFβR-I, Smad2, and Smad4. Scale bar 50 μm. Medial edge epithelium (mee), nasal palatal epithelium (ne) and oral palatal epithelium (oe). **Graph**: comparison of the percentage of epithelial cells positive for Smad2 in E15.5 wild-type and homozygote palates. **b**. Coronal sections through the palate of E14.5 (before fusion) and E15.5 (after fusion) wild-type (WT) and homozygote (*Jf/Jf*) embryos, immunohistochemically stained with pSmad2 antibody. Scale bar 50 μm. Medial edge epithelium (mee). **Graph**: comparison of the percentage of epithelial cells positive for nuclear and cytoplasmic localization of pSmad2 in E15.5 wild-type and homozygote palates. **c**. Coronal sections through the palate of E15.5 wild-type (WT) and homozygote (*Jf/Jf*) embryos, immunohistochemically stained with antibodies against Ki67 and cleaved caspase-3. Scale bar 50 μm. **Graph**: comparison of the percentage of epithelial cells positive for cleaved caspase-3 in E15.5 wild-type and homozygote palates. *P*-values were determined using two-tailed *T*-test.

*Jeff *homozygote palatal shelves start to grow and lift on time, but they fail to fuse at the right developmental stage (Figure 1a) and homozygote mice are born with cleft palate (Figure 1b). This is reminiscent of TGF-β3 mutant mice [29]. To characterize the distribution pattern of TGF-β ligands, TGF-β receptors and Smads in the developing palates, we examined embryonic heads from wild-type and homozygote *Jeff *(*Jf/Jf*) mice by IHC (Figure 2). The result revealed one major difference in the pattern and localization of pSmad2. In wild types at embryonic day 15.5 (E15.5), as the midline epithelial seam was disrupted and medial edge epithelium (MEE) disappeared, pSmad2 was largely confined to the oral/nasal triangle area as a nuclear and cytoplasmic stain (Figure 2b). In contrast, in *Jf/Jf *mice at E15.5 an increased number of epithelial cells are positive for pSmad2 in the palatal shelves concentrated to the tip of the palatal epithelium and the oral/nasal palatal epithelial cells (Figure 2b). Moreover, in *Jf/Jf *palates at E15.5 pSmad2 was present as a nuclear stain in the majority of the cells. In homozygote mice 57% of epithelial cells in the palate showed a nuclear localization of pSmad2, compared with 27% in wild-type mice (*P *= 0.000255). There was a commensurate significant decrease in cells showing a cytoplasmic localization in *Jf/Jf *mice compared with wild-type mice. TGF-β3, TGFβR-I, TGFβR-II, Smad2, Smad4, Smurf2 and also Fbox11, in both wild-type and *Jf/Jf *mice, were localized to the cytoplasm of the epithelial cells (Figure 2a and Additional file 1). There was no difference in the distribution of activated Smad2 in wild-type and homozygote palates before the fusion (E14.5) (Figure 2b).

TGF-β3 has been implicated to inhibit MEE proliferation during palatal fusion [30]. We used Ki67 as a marker for proliferation and examined E14.5 (data not shown) and E15.5 palates using IHC. We were unable to detect any significant differences in staining patterns along the MEE between *Jf/Jf *palatal tissues and wild-type mice (Figure 2c). There are three cellular fates for the MEE: epithelial-mesenchymal transformation, migration and programmed cell death. We used a cleaved caspase-3 antibody to compare cells undergoing apoptosis in E15.5 wild-type and homozygote palates. The results indicate that there are fewer apoptotic cells in *Jf/Jf *E15.5 palates (*P *= 0.017547) (Figure 2c), most likely reflecting the absence of shelf fusion that is followed by MEE disintegration.

### Eyelid expression in Jeff mutants

**Figure 3:**
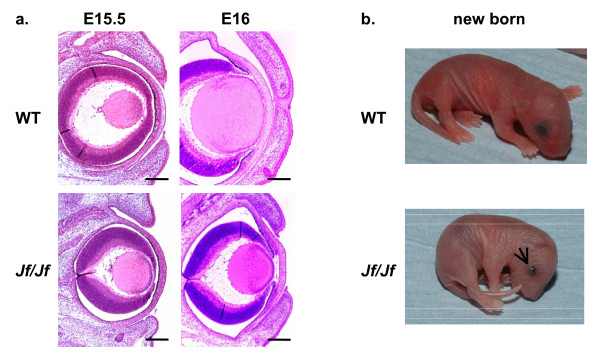
**Eyelid open phenotype**. **a**. Haematoxylin-eosin stained coronal sections through the eyes of E15.5 (before fusion) and E16 (after fusion) in wild-type (WT) and homozygote (*Jf/Jf*) embryos. Scale bars 200 μm. **b**. Failure of eyelids closure in a homozygote mouse (*Jf/Jf*) at birth (arrow).

**Figure 4:**
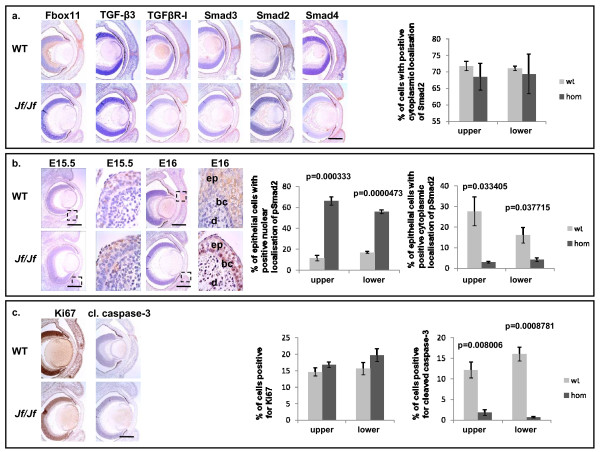
**Immunolocalization in eyelid tissue**. **a**. Coronal sections through the eye of E16 wild-type (WT) and homozygote (*Jf/Jf*) embryos immunohistochemically stained with antibodies against Fbox11, TGF-β3, TGFβR-I, Smad3, Smad2 and Smad4. Scale bar 200 μm. **Graph**: comparison of the percentage of epithelial cells positive for Smad2 in E16 upper and lower eyelids. **b**. Coronal sections through the eyes of E15.5 (before fusion) and E16 (after fusion) in wild-type (WT) and homozygote (*Jf/Jf*) embryos stained with pSmad2 antibody (scale bar 200 μm). Epidermis (ep), basal cells (bc) and dermis (d). **Graph**: comparison of the percentage of epithelial cells with positive nuclear and cytoplasmic localization of pSmad2 in E16 upper and lower eyelids. **c**. Coronal sections through the eyes of E16 in wild-type (WT) and homozygote (*Jf/Jf*) embryos stained with Ki67 and cleaved caspase-3 antibodies. Scale bar 200 μm. **Graph**: comparison of the percentage of epithelial cell positive for Ki67 and cleaved caspase-3 in E16 upper and lower eyelids. *P*-values were determined using two-tailed *T*-test comparing each homozygote eyelid with the wild type.

*Jeff *homozygous mutant eyelids start to grow on time, but fail to fuse at the correct developmental stage and the mice have an EOB phenotype (Figures 3a and 3b). We have applied the same panel of antibodies used on the palates to sections of E16 eyelids from both wild-type and *Jf/Jf *mice. These studies revealed similar results to those observed on palates. The majority of localization of pSmad2 in wild-type E16 eyelids was cytoplasmic. The staining was confined to the epidermis only (Figure 4b). In *Jf/Jf *mice more cells are positive for pSmad2 than in wild type. Moreover, pSmad2 was present as a nuclear stain in the majority of the cells of the epidermis. In homozygote mice 66% of epithelial cells in the upper eyelids and 56% of epithelial cells in the lower eyelids showed nuclear localization of pSmad2, compared with 11% and 17% in the wild-type mice (*P *= 0.000333, *P *= 0.0000473) (Figure 4b). Staining was also observed in the basal layer and also in some cells from the dermis of *Jf/Jf *eyelids (Figure 4b). The expression pattern of TGF-β3, TGFβR-I, TGFβR-II, Smad2, Smad3, Smad4, Smurf2 and Fbox11 at the same stage E16 in both wild-type and *Jf/Jf *eyelids was similar (Figure 4a and Additional file 1). They were all localized to the cytoplasm of the epithelial cells of the epidermis (Figure 4a and Additional file 1). We examined also the distribution of pSmad2 at E15.5, before the closure. In both wild-type and *Jf/Jf *eyelids activated Smad2 was detectable as nuclear and cytoplasmic staining. In homozygote mice 27% of epithelial cells in the upper eyelids and 19% of epithelial cells in the lower eyelids showed nuclear localization of pSmad2, compared with 41% and 32% in the wild-type mice. Eleven per cent of epithelial cells in the homozygote upper eyelids and 9% of epithelial cells in the homozygote lower eyelids showed cytoplasmic localization of pSmad2, compared with 12% and 9% in the wild-type eyelids (data not shown) (Figure 4b).

To compare apoptotic cells in wild-type and *Jf/Jf *junctional zones of the eyelids we again employed cleaved caspase-3 antibody. As with our palate studies, we observed fewer cells positive for cleaved caspase-3 in *Jf/Jf *E16 eyelids (Figure 4c) (*P *= 0.008006 for the upper and *P *= 0.0008781 for the lower eyelids). The process of eyelid closure coordinates both cell proliferation and migration. We used Ki67 as a marker for proliferation, comparing the number of Ki67 positive cells in the epidermis of E16 of wild-type and *Jf/Jf *eyelids. We saw no significant differences, indicating that proliferation is not affected in the *Jf/Jf *eyelids (Figure 4c).

### Lung phenotype of Jf/Jf mice

**Figure 5:**
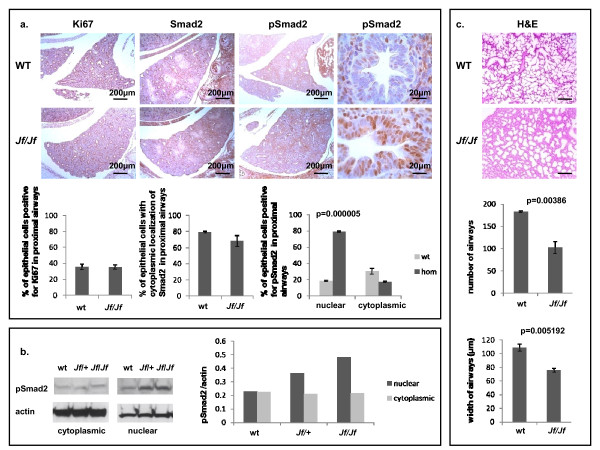
**Lung phenotype**. **a**. Sections through the lungs of E15.5 wild-type (WT) and homozygote (*Jf/Jf*) embryos, immunohistochemically stained with antibodies against Ki67, Smad2 and pSmad2 (scale bars 200 μm and 20 μm as indicated). **Graphs**: comparison of the percentage of cells positive for Ki67, Smad2 and pSmad2 in proximal airways. **b**. Western blot analysis: cytoplasmic and nuclear fractions from wild-type (wt), heterozygote (*Jf*/+) and homozygote (*Jf/Jf*) whole E15.5 lungs. Equal amounts of protein were subjected to tris-acetate PAGE, transferred and probed with pSmad2 and actin antibody for loading control. **Graph**: comparison of the normalised pSmad2 signals in the two fractions. **c**. Haematoxylin-eosin stained sections through new born wild-type (WT) and homozygote (*Jf/Jf*) lungs. Scale bars 200 μm. **Graph**: comparison of the number of airways for three regions of 5.3 × 10^6 ^μm^2 ^taken at random and the width of 3 × 30 airways for each genotype. *P*-values were determined using two-tailed *T*-test.

Newborn *Jf/Jf *mice die soon after birth and gasp for air. Moreover, lung development, like palate and eyelid development, involves growth of epithelial tissue and, in addition, the TGF-β signalling pathway is known to be important in this process. We therefore investigated whether lung development was affected in *Jf/Jf *embryos. Examination of pSmad2 expression in *Jf/Jf *lungs revealed a similar picture to that which we had previously observed in both the eyelids and the palate (Figure 5a). The localization of pSmad2 in E15.5 wild-type lungs was cytoplasmic and nuclear. In *Jf/Jf *lungs the number of positive cells was significantly increased and the localization was exclusively nuclear. The increase in pSmad2-positive cells was particularly striking in proximal airways where 19% of wild-type proximal airway cells were positive for nuclear pSmad2, and this increased to 79% in *Jf/Jf *(*P *= 0.00005); see Figure 5a. In contrast, no significant difference was seen in the localization of Smad2 between wild-type and *Jf/Jf *mice. The accumulation of pSmad2 in the nucleus of whole lungs was also examined by Western blot analysis (Figure 5b) and clearly demonstrated a marked increase in nuclear localization in *Jf/Jf *homozygotes, consistent with our observations from immunostaining. Immunostaining of lungs with a marker for proliferation (Ki67) showed no difference between wild-type and homozygous mutant lungs (Figure 5a).

In newborn *Jf/Jf *mice the lungs are severely affected. We observed thickened interstitial mesenchyme and fewer, smaller alveoli than in wild-type littermates. On average, the airway width in homozygous mutants was significantly smaller than wild-type tissue (Figure 5c). Moreover, quantification of the number of airways indicated a very significant reduction in homozygous mutants (Figure 5c). The mutant mice die soon after birth, probably as a consequence of their lung defect. Cleft palate does not lead to death this early. Immunostaining of lungs with a marker for Clara cells in the proximal airways (CC10) showed no difference in the number or structure of proximal airways between wild-type and homozygous mutant lungs (data not shown).

### Genetic interaction of Fbxo11 and Smad2

**Figure 6:**
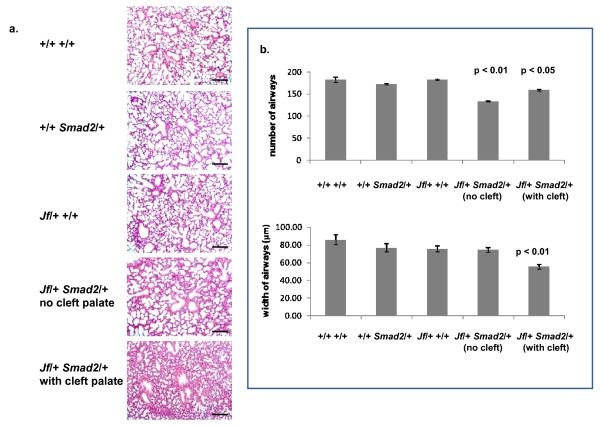
**Lung phenotype of double heterozygotes**. **a**. Sections through the lungs of newborn wild-type (+/+ +/+), heterozygote for Smad2 (+/+ *Smad2*/+), heterozygote for *Jeff *(*Jf*/+ +/+) and two double heterozygote (*Smad2*/+ *Jf*/+) mice: with and without cleft palate. The sections were haematoxylin-eosin stained. Scale bars 200 μm. **b**. Graphic comparison of the number of airways for three regions of 5.3 × 10^6 ^μm^2 ^taken at random and the width of airways for each genotype. *P*-values were determined using two-tailed *T*-test comparing each with the wild type.

It appears that the *Jeff *mutation results in an increased expression as well as increased nuclear localization of pSmad2 in epithelial tissues. We therefore decided to examine genetically the interaction of *Smad2 *and *Fbox11*. We crossed mice heterozygous for a *Smad2 *null mutation to *Jeff *heterozygotes to produce double heterozygous mice: *Jf/+ Smad2/+*. Double heterozygotes comprised 21.6% (57/264) of mice from this cross, significantly different from expected (χ^2 ^= 8.54546, *P *= 0.035986, df = 3). However, 66% (36/57) of *Jf/+ Smad2/+ *mice died soon after birth due to respiratory problems. We performed an extensive histological examination of the palates and lungs of *Jf/+ Smad2/+*, *Jf/+*, *Smad2/+ *and wild-type mice. Notably, *Jf/+ Smad2/+ *mice with early postnatal mortality demonstrated cleft palate and poorly developed lungs, recapitulating the *Jf/Jf *phenotype of smaller and fewer airways (Figure 6). A proportion (34%) of *Jf/+ Smad2/+ *mice survives. These mice do not have cleft palate and appear to display a milder lung phenotype (Figure 6). The surviving double heterozygous mice are also smaller than their wild-type and single heterozygous littermates.

### Genetic interaction of Fbxo11 and p53

Recently, it has been shown that Fbxo11 acts as a Nedd8-ligase to p53. Neddylation of p53 by Fbxo11 leads to a reduction in transcriptional activity [14]. Given these findings and that p53 is a co-factor of pSmad2, we examined the genetic interaction between p53 and Fbxo11. *Jf/+ *mice and p53 homozygotes were intercrossed to produce double heterozygotes – *Jf/+ p53/+*. Compound heterozygotes comprised 44.9% (31/69) of the mice born from this cross (χ^2 ^= 0.72464, *P *= 0.399397, df = 1). However, all *Jf/+ p53/+ *mice appeared phenotypically normal (data not shown).

### Biochemical interactions of Fbxo11, Smad2 and p53

**Figure 7:**
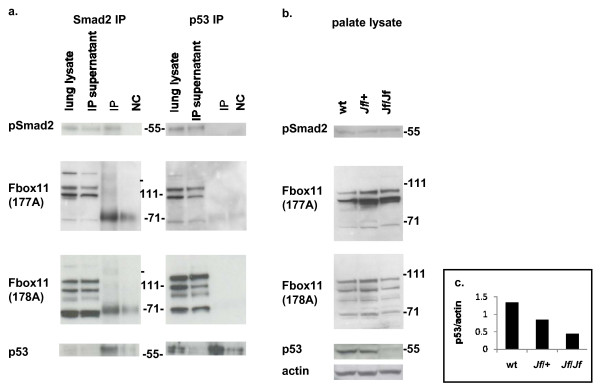
**Immunoprecipitation and Western blot analysis**. **a**. Immunoprecipitation: lung extract from E15.5 wild-type embryos was used for immunoprecipitation using Smad2 and p53 antibodies. The Western blots were probed with pSmad2, Fbox11 and p53 antibodies. **b**. Western blot analysis: protein lysates from wild-type, heterozygote and homozygote E15.5 palates. Equal amounts of protein were subjected to tris-acetate PAGE, transferred and probed with pSmad2, Fbox11, p53 antibodies and actin antibody for loading control. **c**. Graphical representation of p53 levels normalized against actin in wild-type, heterozygote and homozygote E15.5 palates, showing approximately threefold reduction of p53 in homozygote palate compared with wild type.

We explored further the genetic interaction between Fbxo11 and Smad2 by performing immunoprecipitations to test whether these two proteins interact. We used a cross-linking agent to improve the likelihood of detecting the interactions. However, immunoprecipitations with Smad2 antibodies failed to reveal any interaction with Fbxo11 (Figure 7a), suggesting that Smad2 or pSmad2 is not a substrate for ubiquitination by Fbxo11. However, Smad2 did immunoprecipitate p53, confirming the known interaction between these two proteins (Figure 7a).

We also undertook immunoprecipitations with p53 (Figure 7a) using lung tissue. We also failed to detect any interaction between Fbxo11 and p53, despite the reported neddylation of p53 by Fbxo11. Intriguingly, we find that in *Jeff *homozygote mice p53 is expressed at very low levels (Figure 7b and 7c). The heterozygote appears to display intermediate levels. These results suggest that Fbxo11 plays a role in stabilising p53.

## Discussion

We previously identified a mutation in *Fbxo11 *in the *Jeff *mutant mice [8]. *Jeff *heterozygote mice develop chronic OM [7] and *Jeff *homozygotes demonstrate perinatal lethality, cleft palate and an EOB phenotype [8]. We investigated the impact of the *Jeff *mutation on the TGF-β signalling pathway, paying particular attention to those members of the pathway that have a role in epithelial development.

In mammalian development the formation of the palate is a multi-step process, involving palatal shelf growth and elevation above the tongue, followed by fusion of the shelves and the disappearance of the MEE [31]. Defects in any of these steps can lead to cleft palate, one of the most common birth defects in humans. Murine palatogenesis takes place between E11.5 and E15.5 [31]. All TGF-β ligands are known to be expressed at that stage in the developing palate [32]. Amongst them, TGF-β3 is likely to be the most important in palatogenesis since mutation of the gene causes cleft palate in mice [29,33] and humans [34]. In palate cultures from these mutant embryos, the fusion defect is rescued by endogenous TGF-β3 [35]. It has been demonstrated that TGF-β3 selectively regulates the disappearance of MEE during palatal fusion: TGF-β3 disintegrates the MEE basement membrane [36]. Studies on the mechanism for MEE disappearance during palatal fusion suggests a role of TGF-β3 as an inducer of apoptosis – cell death in TGF-β3 null palates is reduced at the time of fusion [37]. Smad2 and Smad3, mediators of TGF-β signalling, are expressed in the MEE cells, but only Smad2 is phosphorylated during palatal fusion. Smad2 phosphorylation is temporo-spatially restricted to the MEE and correlates with the disappearance of the MEE [30]. TGF-β3 is required for phosphorylation of Smad2 in the MEE and the inhibition of MEE proliferation during palatal fusion [30].

Our studies using IHC to co-localize the TGF-β3 ligand, TGF-β receptors and Smads in developing palates revealed one major difference between wild-type and *Jf/Jf *mice. At E15.5 more epithelia cells are positive for pSmad2 in the homozygote palatal shelves than in the wild type. Moreover, we found that there was a substantial increase in nuclear localization of pSmad2 in *Jf/Jf *mice. This might suggest that the turnover of pSmad2 is stalled and pSmad2 accumulates in the nucleus, with consequent effects for TGF-β signalling in the epithelia.

TGF-β3 plays a role as an inhibitor of MEE proliferation during palatal fusion [30] and an inducer of apoptosis [37]. Using Ki67 as a marker for proliferation we did not observe increased proliferation in E14.5 and E15.5 palates of the *Jeff *homozygote MEE. However, we also stained for apoptotic cells using a caspase-3 antibody and found many fewer positive cells in *Jeff *homozygote E15.5 palates. It appears that in the presence of a mutation in *Fbxo11*, palatal fusion is inhibited and MEE cells are not able to progress to epithelial programmed cell death or epithelial-mesenchymal transformation.

The palatal shelves fusion is a permanent fusion, such as the fusion of the neural tube. During mammalian development some temporary fusions also occur for example, eyelid fusion, fusion of the digits and fusion of the pinnae of the ears to the scalp. The disjunction of the temporary fusions takes place after birth [38]. The eyelids develop at approximately the same time as the palatal shelves. They start to form at about E11.5, grow across the eye from E14 to E16 and, as the fusion progresses, the diminishing gap fills with a profusion of rounded cells that are extruded, flattened, and sloughed off from the area of completed fusion [38,39]. Failure of the eyelids to grow and fuse in mice leads to the EOB defect. Mouse knockout studies have identified several signalling molecules involved in the control of embryonic eyelid closure and some of them are from the TGF-β family. Mutation in activin βB results in an EOB phenotype [40] similar to that observed in MEKK1-deficient mice [41]. TGF-β/activin signalling via the MEKK1-mediated JNK pathway is one pathway controlling eyelid closure [17,42]. MEKK1 is not required however for the classical TGF-β/activin pathway, which involves nuclear translocation of Smad proteins [17].

Normally in mouse development, the eyelid fusion is complete by E16, but the eyelids of *Jeff *homozygote mice remain wide apart. The eyelids start to form the leading edge of the developing margins, but the leading edge is not extended and the eyelids do not reach each other. We applied the same panel of antibodies, employed on the palates, on sections of E16 eyelids. The expression pattern of TGF-β3 and the signalling receptor TGFβR-I are consistent with localization observed in the cytoplasm of the cells of the epidermis of both the developing wild-type and mutant eyelids. The distribution of non-activated Smads is also similar, but with pSmad2 there is a clear difference between the wild-type and homozygote eyelids. Activated Smad2 was present as a nuclear stain in the majority of the cells of the epidermis, the basal layer and also some cells from the dermis of the *Jeff *homozygote eyelids. In contrast, in wild-type eyelids the pSmad2 is localized in the cytoplasm of the epidermis. The difference is also time specific. At E15.5 before eyelid fusion the pattern and the localization of activated Smad2 is similar in wild type and *Jf/Jf*. These findings mirror and are consistent with the expression changes observed in the palate.

In the developing eyelid, as with the palate, we also studied proliferation and apoptosis in the *Jeff *homozygote. Using Ki67 as a marker for proliferation, we found that there was little difference between wild-type and mutant homozygotes in the number or distribution of cells stained. However, we found significantly lower staining with caspase-3 in the *Jeff *homozygote, indicating reduced rates of apoptosis. It would appear that, as with the palate, the absence of epithelial fusion prevents the transition to epithelial programmed cell death.

We show here that, in addition to palatal and eyelid development defects, the development of the lung in *Jeff *homozygotes is severely compromised. In the mouse, early lung development begins at about E9.5 with the formation of the paired lung buds and continues through the pseudoglandular phase at E9.5-16.5 when the bronchial and respiratory tree develops, and the canalicular phase E16.5-17.5 when vascularization occurs. During late lung development at E17.5-P4 the distal airways form saccular units (saccular stage) and the secondary septae divides these units during the alveolar stage at P5-28 [43,44].

Several studies have implicated TGF-β signalling in early lung development. Immunohistochemical studies on localization of the three TGF-β isoforms in the developing mouse embryo suggest that they play an important role in lung formation [18]. TGF-β signalling is implicated in the negative regulation of lung growth and development during early lung organogenesis [45]. Recent expression data also indicates that active TGF-β signalling is required for normal late lung development [46]. Gene-targeting studies also demonstrate the involvement of the TGF-β family in early and late lung morphogenesis. TGF-β3 null newborn mice have under-developed and poorly inflated lungs [33]. The developmental delay is detectable as early as day E12.5, which implicates a role of TGF-β3 in early lung development. Retarded lung alveolarization and subsequent emphysema in Smad3 knockout mice, on the other hand, suggests a role for the TGF-β/Smad3 pathway in postnatal lung growth and emphysema prevention [47]. As the TGF-β family is involved in lung morphogenesis and reflecting the changes in pSmad2 expression and localization in the palate and eyelid, we have explored pSmad2 in the developing lung. Again, we find a significant increase in the expression of pSmad2. Most notably, there is a very marked increase in localization of pSmad2 to the nuclei of epithelial cells lining the airways.

Given the effects of the *Fbxo11 *mutation on pSmad2 in a variety of tissues, we decided to examine genetically the interaction between these two genes and generated *Jf/+ Smad2/+ *compound heterozygotes. Intriguingly, we found that *Jf/+ Smad2/+ *mice recapitulated many of the features of *Jeff *homozygotes, including the cleft palate, lung phenotype and post-natal mortality. The phenotypes of the *Jf/+ Smad2/+ *compound heterozygote are not fully penetrant. Around one-third of *Jf/+ Smad2/+ *mice survive but are much smaller than wild-type or single-mutant littermates. The developmental abnormalities observed in the *Jf/+ Smad2/+ *mice contrasts with the normal phenotype seen in *Jf/+ *mice and underlines the effects of the *Fbxo11 *mutation on pSmad2. It would appear that a complete absence of Fbxo11 function, with consequent effects on pSmad2 expression and localization, can lead to the epithelial developmental defects that we have observed. Alternatively, compromising levels of gene expression at both the *Fbxo11 *and *Smad2 *loci leads to the same phenotype.

Despite the effects of the *Fbxo11 *mutation, and the genetic interaction observed between Fbxo11 and Smad2, we failed to observe any biochemical interaction between Fbxo11 and Smad2. As expected we were able to demonstrate the known interaction between Smad2 and p53. It has been reported that Fbxo11 neddylates p53, inhibiting its transcriptional activity [14]. However, immunoprecipitations did not reveal an interaction in the tissues we tested. Indeed, *Jf/+ p53/+ *compound heterozygotes did not show any developmental phenotypes. Nevertheless, we found that *Jeff *homozygotes have markedly reduced levels of p53 and heterozygotes appeared to have intermediate levels. It appears that Fbxo11 is required for stabilisation of p53.

p53 is required for TGF-β responses through its interactions with Smads [15]. p53-deficient mammalian cells demonstrate impaired responses to TGF-β signals. Most importantly, p53 and Smad2 cooperated synergistically at target promoters for TGF-β signalling, and indeed they directly interact *in vivo *in a TGF-β-dependent fashion [15]. We propose that in the Jeff homozygous mutant, loss of *Fbxo11 *function leads to destabilisation of p53 by an unknown mechanism. It is possible, given the synergistic interaction between p53 and Smad2, that the loss of p53 at transcriptional targets leads to stalling and accumulation of pSmad2 in the nuclei of epithelial tissues of *Jeff *homozygotes. Mutant p53 leads to attenuation of TGF-β1 signalling including a reduction in Smad2/3 phosphorylation, inhibition of Smad2/Smad4 complex formation and Smad4 translocation to the nucleus [48]. Overall, given the interaction between p53 and TGF-β signalling pathways, it seems likely that the effects of the *Fbxo11 *mutation on TGF-β signalling may be mediated through effects on p53.

Chronic and recurrent OM in the human population is known to have a very significant genetic component, yet little is known about the underlying genes or pathways involved. Moreover, although a number of mouse strains are available that show OM, they are complicated by poor penetrance or the complex syndromic nature of the disease. *Jeff *heterozygotes develop a highly penetrant chronic suppurative OM in the absence of any other significant pathology [7,8] and thus the *Jeff *mutant represents a powerful model for studying the genetic and pathophysiological bases of chronic/recurrent OM. The discovery of the *Fbxo11 *gene underlying the Jeff mutant identified an important candidate for the study of OM in the human population. Indeed, initial studies with FBXO11 SNPs in human OM families have demonstrated nominal evidence of association, indicating the genetic involvement of human FBXO11 with chronic and recurrent OM [49].

## Conclusion

The studies reported here support a role for Fbxo11 in TGF-β signalling and suggest that perturbations in this pathway may underlie chronic inflammation in the middle ear. This is particularly pertinent given the studies of a similar mouse mutant with chronic OM, *Junbo *[9], caused by a mutation in the transcription factor Evi1 which is a co-repressor of Smad3. Overall, there is increasing evidence that defects in TGF-β signalling or associated pathways may underlie the development of chronic OM, and genes in these pathways represent good candidates for future human association studies.

## Methods

### Mice

The *Jeff*, *Smad2 *and *p53 *colonies were maintained on the C57BL/6J background and genotyped as previously described [8,50,51].

### Histology

The embryonic (E14.5-E16) and newborn heads and bodies were fixed in 10% buffered formaldehyde, decalcified and embedded in paraffin following routine procedures. The lungs of the newborn mice were inflated with 10% buffered formaldehyde, fixed and paraffin-embedded. Three-micrometer-thick sections were obtained, de-paraffinized in xylene substitute and rehydrated via a graded ethanol. For morphological observations, sections were stained with haematoxylin and eosin.

### Antibodies

The antibodies were as follows: rabbit polyclonal TGF-β3 (sc-90, Santa Cruz Biotechnology), rabbit polyclonal TGFβR-I (sc-398, Santa Cruz Biotechnology), rabbit polyclonal TGFβR-II (ab28382, Abcam), goat polyclonal Smad2 (sc-6200 Santa Cruz Biotechnology), rabbit polyclonal Smad3 (06-920 Upstate), rabbit polyclonal Smad4 (06-693 Upstate), rabbit polyclonal anti-phospho Smad2 (Ser 465/467) (AB3849 Chemicon International and 3101 Cell Signaling), rabbit polyclonal SMURF2 (07-249 Upstate), rabbit polyclonal FBXO11 (A301-177A, A301-178A Bethyl Laboratories), rabbit polyclonal Fbxo11 produced by Covalab UK [8], rabbit polyclonal p53 (sc-6243 Santa Cruz Biotechnology), agarose-conjugated mouse monoclonal p53 (sc-126 AC), rabbit polyclonal Ki67 (VP-K451 Vector Laboratories), rabbit polyclonal cleaved caspase-3 antibody (9661 Cell Signaling Technology).

### Immunostaining

For immunohistochemical analysis, the avidin-biotin complex (ABC) method was used for all the antibody stainings except for the TGF-β3 antibody. Endogenous peroxidase activity was quenched with 3% hydrogen peroxide in isopropanol for 20 min. Slides for Ki67, cleaved caspase-3, TGFβ-RII and SMURF2 antibodies were pre-treated by boiling in a microwave in 10 mM sodium citrate buffer pH 6 for 14 min. Slides for Fbxo11 were pre-treated by boiling in a microwave in water for 14 min. After pre-treatment the slides were cooled at room temperature for 20 min and rinsed with phosphate-buffered saline. To inhibit non-specific endogenous biotin staining, the DAKO Biotin Blocking System was used (DAKO, X0590). Rabbit ABC Staining system (sc-2018 Santa Cruz Biotechnology) and goat ABC staining system (sc-2023 Santa Cruz Biotechnology) were used to develop the specific signals with all the antibodies except for TGF-β3. The antibody incubations were as follow: TGFβ-RII 1:100 dilution and SMURF2 1:200 dilution for 30 min; Fbxo11 1:200 dilution for 1 h, TGFβ-RI, Smad2, pSmad2 (Ser 465/467), Smad3 and Smad4 overnight using a 1:200 dilution; Ki67 was incubated for 1 h at room temperate using a 1:1000 dilution, and cleaved caspase-3 for 1 h in 1:200 dilution. The slides were counterstained with haematoxylin.

For the immunohistochemical analysis with TGF-β3 antibody, slides were blocked with 5% bovine serum albumin, incubated with the antibody overnight using a 1:200 dilution, washed with phosphate-buffered saline, and an anti-rabbit FITC-conjugated antibody (Sigma, F7512) was used as a secondary antibody for 30 min at room temperature in 1:160 dilution. Sections were washed with phosphate-buffered saline, mounted in Vectashield mounting medium (Vector Laboratories) and the staining was observed by fluorescence microscopy. Negative control sections were incubated with serum instead of the antibody and otherwise processed identically.

### Immunoprecipitation

Protein samples from lungs of E15.5 wild-type embryos were prepared by homogenizing in phosphate buffer solution, containing 1% triton X100, 0.5% deoxycholic acid and cocktail of protease inhibitors. The samples were incubated with 2 mM 3,3'-Dithiobis [sulfosuccinimidylpropionate] (DTSSP) cross-linker for 30 min at room temperature and treated as described by the manufacturer (PIERCE, 21578). Total protein extract of 1 mg was pre-cleared with 15 μl Sepharose-protein G beads (Sigma) for 1 h at 4°C with rotation. The pre-cleared extract was incubated with 4 μg of antibody (Smad 2, p53) overnight at 4°C. Twenty microlitres of protein G beads was added and allowed to bind for 3 h at 4°C with rotation. Beads were washed four times with 500 μl of extraction buffer and resuspended in electrophoresis loading buffer/reducing agent (NuPAGE, Invitrogen). As a negative control, an identical reaction was prepared where goat IgG (for Smad2) or mouse IgG (for p53) was used instead of the specific antibody. Cross-linking reversal was achieved by incubation at 37°C for 30 min, after which samples were heated at 70°C for 10 min and resolved in NuPAGE 7% Tris-Acetate gels (Invitrogen). Only one third of the total reaction was loaded per lane.

### Nuclear and cytoplasmic fractionation

For the fractionation CelLytic NuCLEAR Extraction kit (Sigma, NXTRACT) was used. E15.5 lungs were lysed in 1× lysis buffer containing protease and phosphatase inhibitors using a glass homogenizer. Nuclei were pelleted by centrifugation and the cytoplasmic fraction obtained by retaining the supernatant. The nuclear extracts were obtained by resuspending the nuclei pellet in extraction buffer containing protease and phosphatase inhibitors, rocking the pellet for 30 min and subsequent centrifugation. Twenty micrograms of each protein sample was loaded on each lane.

### Western blot

Gels were blotted onto PVDF (GE Healthcare) and blocked in phosphate-buffered saline containing 5% dry skimmed milk and 0.1% Tween 20. Antibodies were diluted in blocking solution at 1:250 (anti-p53), 1:500 (anti-pSmad2), 1:500 (anti-FBOX11) or 1:10,000 (anti-rabbit-HRP). Incubation with primary antibodies proceeded overnight at 4°C and with secondary antibody for 1 h at room temperature. Membranes were washed four times between antibodies and after secondary antibody in phosphate-buffered saline and 0.1% Tween 20. ECL Plus (GE Healthcare) was used as detection system.

### Data analysis

We used chi-squared test to compare the difference between the observed and the expected number of the double heterozygous mice from the two crosses. To evaluate the probability of the calculated chi-squared value the chidist function in Excel was used.

## Competing interests

The authors declare that they have no competing interests.

## Authors' contributions

HT carried out the immunostaining analysis, some of the proteomics analysis, contributed to the design of the study and the interpretation of the results and participated in drafting the manuscript. RH contributed to the analysis and discussion of the results. SM participated in the phenotyping and the genotyping of some of the mice, and MRR and HH performed most of the immunoprecipitations and western blot analysis and contributed to the interpretation of the results from these studies. CD helped with the study of the lung phenotype of the mice and contributed to the interpretations of the results. SDMB contributed to the design of the study, interpretation of results and participated in drafting the manuscript. All authors read and approved the final manuscript.

## Supplementary Material

Additional file 1 **Immunolocalization of TGFβR-II and Smurf2**. Sections through E15.5 palate, E16 eyelids and E15.5 lungs of wild-type (WT) and homozygote (*Jf/Jf*) embryos, immunohistochemically stained with antibodies against TGFβR-II (upper panel) and Smurf2 (lower panel). Scale bars 20, 50 and 200 μm as indicated. Click here for file
