# Supplementary figures and images for: Regulation of TGF-β signalling by Fbxo11, the gene mutated in the Jeff otitis media mouse mutant
Source: Pathogenetics. 2009 Jul 6;2:5. doi: 10.1186/1755-8417-2-5 (PMC2714483; doi:10.1186/1755-8417-2-5)

## Slide 1
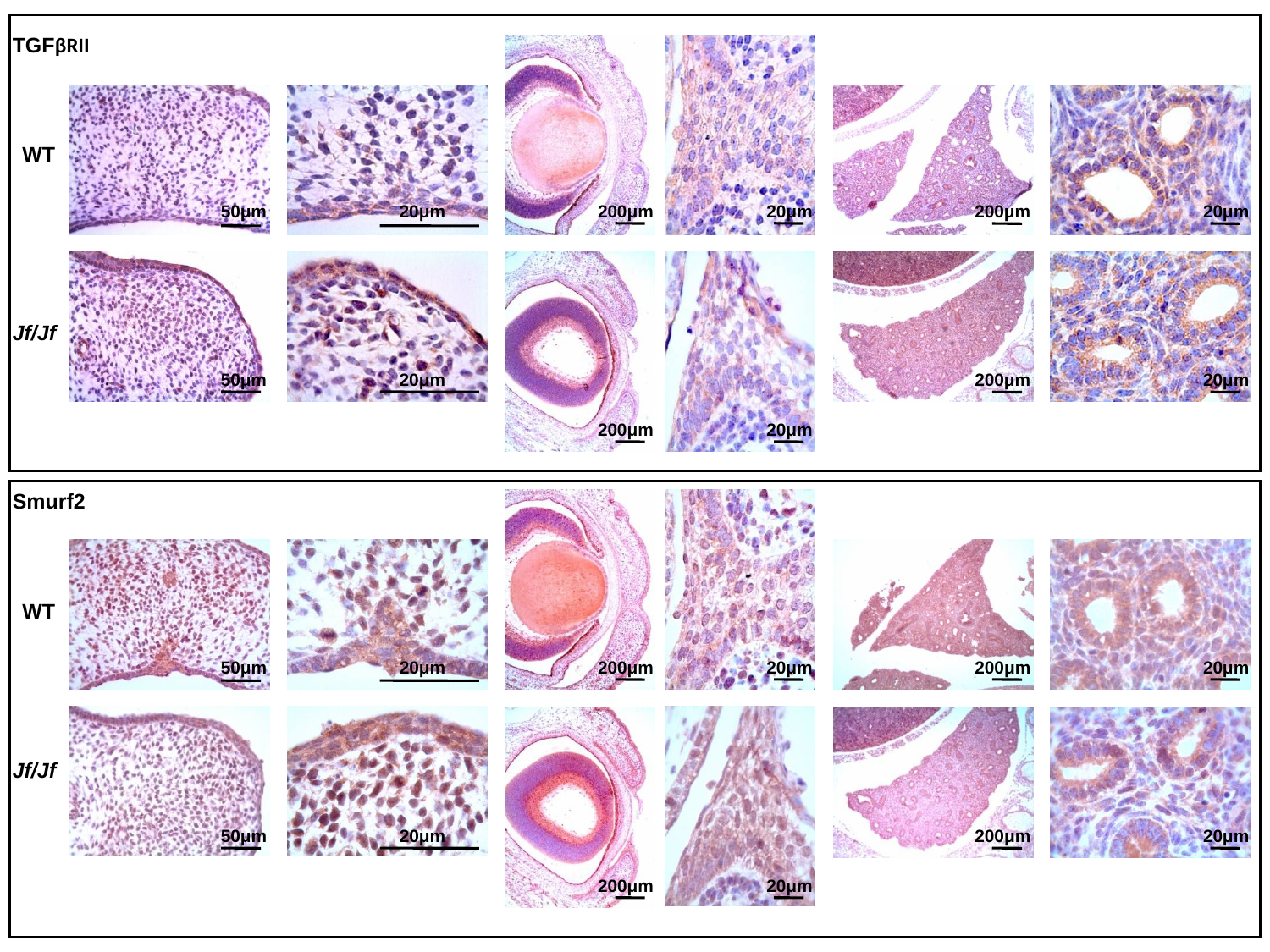

TGFβRII
WT
50μm
20μm
200μm
20μm
200μm
20μm
Jf/Jf
50μm
20μm
200μm
20μm
200μm
20μm
Smurf2
WT
50μm
20μm
200μm
20μm
200μm
20μm
Jf/Jf
50μm
20μm
200μm
20μm
200μm
20μm

Supplement: Additional file 1 — Immunolocalization of TGFβR-II and Smurf2. Sections through E15.5 palate, E16 eyelids and E15.5 lungs of wild-type (WT) and homozygote (Jf/Jf) embryos, immunohistochemically stained with antibodies against TGFβR-II (upper panel) and Smurf2 (lower panel). Scale bars 20, 50 and 200 μm as indicated. [file 1755-8417-2-5-S1.ppt]
